# Tissue-specific bioactivity of soluble tendon-derived and cartilage-derived extracellular matrices on adult mesenchymal stem cells

**DOI:** 10.1186/s13287-017-0580-8

**Published:** 2017-06-05

**Authors:** Benjamin B. Rothrauff, Guang Yang, Rocky S. Tuan

**Affiliations:** 1 Center for Cellular and Molecular Engineering, Department of Orthopaedic Surgery, University of Pittsburgh School of Medicine, 450 Technology Drive, Room 221, Pittsburgh, PA 15219 USA; 2 McGowan Institute for Regenerative Medicine, University of Pittsburgh, Pittsburgh, PA 15219 USA; 3 Department of Bioengineering, University of Pittsburgh Swanson School of Engineering, Pittsburgh, PA 15219 USA

**Keywords:** Decellularized extracellular matrix, Tendon, Cartilage, Differentiation

## Abstract

**Background:**

Biological scaffolds composed of tissue-derived extracellular matrix (ECM) can promote homologous (i.e., tissue-specific) cell differentiation through preservation of biophysical and biochemical motifs found in native tissues. Solubilized ECMs derived from decellularized tendon and cartilage have recently been promoted as tissue-specific biomaterials, but whether tissue-specific bioactivity is preserved following solubilization is unknown. This study explored the tissue-specific bioactivity of soluble decellularized tendon and cartilage ECMs on human bone marrow-derived mesenchymal stem cells (MSCs) presented across different culture microenvironments, including two-dimensional (2D) tissue culture plastic, aligned electrospun nanofibers, cell pellets, and cell-seeded photocrosslinkable hydrogels.

**Methods:**

Tendon and cartilage ECMs were decellularized using established methods and solubilized either via pepsin digestion or urea extraction. The effect of soluble ECMs on cell proliferation and differentiation was initially explored by supplementing basal medium of human MSCs cultured on 2D tissue culture plastic. In subsequent experiments, MSCs were cultured on aligned electrospun nanofibers, ascell pellets, or encapsulated within photocrosslinkable methacrylated gelatin (GelMA) hydrogels. Urea-extracted tendon and cartilage ECMs were added as supplements.

**Results:**

Pepsin-digested ECMs did not promote homologous differentiation in human MSCs, whether provided as a medium supplement or three-dimensional (3D) hydrogels. In contrast, urea-extracted ECMs tended to promote tissue-specific differentiation of MSCs cultured in 2D and 3D microenvironments. The application of the small molecule TGF-β signaling inhibitor SB-431542 largely negated the tissue-specific gene expression patterns mediated by tendon and cartilage ECMs. This suggests that the action of endogenous TGF-β was required, but was not sufficient, to impart tissue-specific bioactivity of urea-extracted ECMs. When urea-extracted cartilage ECM was incorporated within a photocurable GelMA hydrogel it independently enhanced chondrogenesis in encapsulated MSCs, and showed additive prochondrogenesis upon TGF-β supplementation in the medium.

**Conclusions:**

Urea-extracted ECM fractions of decellularized tendon and cartilage are soluble supplements capable of enhancing tissue-specific differentiation of adult stem cells.

**Electronic supplementary material:**

The online version of this article (doi:10.1186/s13287-017-0580-8) contains supplementary material, which is available to authorized users.

## Background

Injuries to tendon and cartilage present a persistent clinical challenge given the poor intrinsic healing capacity of these tissues. Tissue engineering strategies, which employ the independent or combined application of cells, scaffolds, or biomolecules, have shown promise in restoring the structure and function of both tendon [1, 2] and cartilage [3, 4]. Biomimetic scaffolds, including aligned electrospun nanofibers [5, 6] and hydrogels [7, 8], possess ultrastructural motifs respectively found in native tendon and cartilage which are capable of directing differentiation of mesenchymal stem cells (MSCs) towards a particular musculoskeletal lineage. These effects are further enhanced by exposure to soluble biomolecules known to orchestrate tendon and cartilage development [9–11]. In particular, the transforming growth factor beta (TGF-β) superfamily plays an essential role in both tenogenesis [12] and chondrogenesis [13], mediating divergent effects depending upon additional microenvironmental cues [14]. At present, an incomplete understanding of tendon and cartilage development and homeostasis limits tissue engineering approaches to regenerate these tissues [15].

On the other hand, tissues and organs can be decellularized to mitigate an adverse immune response against foreign cells while theoretically preserving the ultrastructural, mechanical, and biochemical motifs of the native tissue [16]. To that end, decellularized tendon and cartilage may serve as the ideal scaffold to promote homologous (i.e., tissue-specific) differentiation of endogenously recruited or exogenously delivered progenitor cells [17, 18]. Indeed, decellularized tendon [19–21] and cartilage [22, 23] tissues have been found to promote tissue-specific differentiation when seeded with MSCs. Nevertheless, the dense collagenous architecture comprising the extracellular matrix (ECM) of these tissues can necessitate the use of relatively harsh decellularization methods to sufficiently remove native cells which can compromise native tissue ultrastructure and biochemical composition. Even with sufficient removal of cellular content, the dense ECM serves as a barrier for cell infiltration, with cells often localized to the tissue surface [23, 24]. In addition, the use of whole decellularized tissue as grafts requires surgical reconstruction/transplantation (as opposed to repair), with resulting limitations in treating small or irregularly shaped defects.

In an effort to overcome these limitations while retaining the tissue-specific bioactivity inherent in the ECM, decellularized tissues have been processed into powders, which can be molded into distinct geometric shapes [25, 26], or suspended in a hydrogel [27–29]. Alternatively, ECM powders can be solubilized with enzymatic or chaotropic agents, resulting in an injectable solution that can be combined with a diverse array of biomaterials. Pepsin-digested tendon [30, 31] and cartilage [32, 33] hydrogels have been shown to undergo thermoresponsive gelation at body temperature and are cytocompatible. However, the effect of pepsin, a nonspecific protease, on the tissue-specific bioactivity of tendon and cartilage ECM remains unknown. While Keane et al. [34] reported that pepsin-digested esophageal ECM hydrogels supported esophageal stem cell migration and organoid formation to a greater extent than heterologous ECM hydrogels, Lin et al. [35] found that a pepsin-digested extract of decellularized MSC sheets provided no additional benefit over collagen (COL) type I hydrogels; conversely, a urea-extracted fraction enhanced MSC attachment, spreading, proliferation, migration, and multi-lineage differentiation. Similarly, Zhang et al. [36] and Yang et al. [37] independently reported that urea-extracted fractions of decellularized ECM from diverse tissues were capable of promoting tissue-specific differentiation.

In this study, soluble decellularized tendon and cartilage ECMs were initially prepared by pepsin digestion or urea extraction. As early experiments with two-dimensional (2D) cultures suggested the superiority of urea-extracted over pepsin-digested solutions in terms of supporting proliferation and tissue-specific differentiation of MSCs, the bioactivities of urea-extracted ECM solutions were further investigated across several three-dimensional (3D) conditions commonly employed for cell-based tissue engineering and regeneration, including electrospun nanofibers, pellet culture, and photocrosslinked methacrylated gelatin (GelMA) hydrogels. Supplementation of culture medium with TGF-β3 served as a positive control. We hypothesized that urea-extracted ECM fractions would promote tissue-homologous differentiation of MSCs under multiple 3D culture conditions.

## Methods

### Overview

Tendon and hyaline cartilage were procured from bovine hindlimbs and subsequently decellularized and characterized. Tendon and cartilage ECMs were then solubilized through either acid-pepsin digestion (tAP and cAP, respectively) or urea extraction (tECM and cECM, respectively), and their respective effects on human MSC proliferation and gene expression were determined in 2D culture. MSCs were cultured as pellets, seeded on aligned nanofibrous scaffolds, or encapsulated in photocrosslinked GelMA hydrogels, and exposed to media supplemented with TGF-β3, urea-extracted tendon ECM (tECM), or urea-extracted cartilage ECM (cECM). Assays for gene expression, histology, and biochemical composition were performed to assess tissue-specific bioactivities of the supplements. Additionally, the effect of inhibiting endogenous TGF-β present in urea-extracted ECM fractions, using the small molecule inhibitor SB-431542, was assessed using pellet cultures.

### Decellularization of tendon and cartilage

Patella tendon and articular cartilage were procured from hindlimbs of 6–8 week old cows (Research 87, Boylston, MA, USA) and stored at −20 °C in a protease inhibitor solution composed of phosphate-buffered saline (PBS; Gibco, Grand Island, NY, USA) supplemented with 5 mM ethylenediaminetetraacetic acid (EDTA; Sigma-Aldrich, St. Louis, MO, USA) and 0.5 mM phenylmethylsulfonyl fluoride (PMSF; Sigma-Aldrich) until use. Upon thawing, tissues were minced (8–27 mm^3^) and cryomilled (Spex Freezer Mill 8670, Metuchen, NJ, USA). Four grams of wet tissue powder were suspended in 40 mL of protease inhibitor solution containing 1% Triton X-100 (Sigma-Aldrich) and agitated for 24 h at 4 °C, followed by three washes for 30 min each in PBS. Tissue powders were subsequently exposed to 40 mL of Hank’s buffered salt solution (HBSS; ThermoFisher Scientific, Pittsburgh, PA, USA) supplemented with 200 U/mL DNase and 50 U/mL RNase (Worthington, Lakewood, NJ, USA) for 12 h at room temperature. Decellularized powders were then washed six times with PBS and characterized for histological appearance and biochemical composition.

### Solubilization of decellularized ECM

#### Pepsin digestion

Decellularized tendon and cartilage ECM powders were enzymatically digested in a solution of 1 mg/mL porcine pepsin (Sigma-Aldrich) in 0.01 N HCl for 48 h at room temperature under continuous stirring. For use as a medium supplement, digested tendon and cartilage ECM were neutralized by the addition of one-tenth digest volume of 0.1 N NaOH and one-ninth digest volume of 10× PBS while keeping the samples at 4 °C. To form 3D hydrogels, pH-neutralized digests were warmed to 37 °C for 1 h, as reported previously [34, 38].

#### Urea extraction

A water-soluble fraction of tendon and cartilage ECM was obtained through urea extraction, as previously described [37]. Briefly, 4 g of wet decellularized ECM powder was agitated for 3 days at 4 °C in 40 mL of 3 M urea dissolved in water. The suspension was centrifuged for 20 min at 1500 g and the supernatant was transferred to benzoylated tubing (Sigma-Aldrich) and dialyzed against ddH_2_O for 2 days at 4 °C, changing the water every 8 h. The dialyzed ECM extract was transferred to centrifugal filter tubes (3000 MWCO; EMD Millipore, Billerica, MA, USA) and spin-concentrated approximately 10-fold at 1500 g for 60 min. The final ECM extract was filter-sterilized through a PVDF syringe filter unit (0.22 μm; EMD Millipore). Protein concentration was determined by BCA assay (ThermoFisher Scientific) and aliquots of 1 mg/mL were stored at −80 °C until further use. Before use in experimental studies, aliquots prepared from three different batches were pooled.

### SDS-PAGE and growth factory analysis of soluble ECM

Samples of native tendon and cartilage ECMs, and their corresponding urea-extracted and pepsin-digested extracts, were suspended in TM buffer (Total Protein Extraction Kit, EMD Millipore); 30 μg total protein was mixed with SDS loading buffer and reducing agent (NuPAGE; Life Technologies, Carlsbad, CA, USA) and heated for 10 min at 70 °C. Protein was loaded into a pre-cast 10-well NuPAGE 4–12% Bis-Tris Minigel (Life Technologies) and separated by electrophoresis in MOPS running buffer for 50 min at constant 200 V. The gel was stained with SimplyBlue™ SafeStain (Life Technologies) for 1 h, washed several times in water, and photographed using a CCD camera gel imaging system (FOTODYNE, Hartland, WI, USA).

Additionally, the growth factor contents of the soluble ECM preparations were determined using a Human Growth Factor Array (RayBiotech, Norcross, GA, USA) according to the manufacturer’s instructions.

### MSC isolation

Human MSCs were obtained as previously described [35], with Institutional Review Board approval (University of Washington and University of Pittsburgh). MSC populations isolated from individual patients were routinely validated as capable of osteogenic, adipogenic, and chondrogenic differentiation (data not shown). All experiments were performed with passage 3 (P3) MSCs. MSCs from three patients (56-year-old male, 56-year-old female, 59-year-old male) were pooled for this study.

### Bioactivity of soluble ECM in two-dimensional cell culture

To determine the effect of soluble ECM preparations on MSC morphology, proliferation, and metabolism, 1 × 10^3^ cells/cm^2^ were suspended in growth medium (Dulbecco’s modified Eagle’s medium (DMEM), 10% v/v fetal bovine serum (FBS), 1% v/v penicillin-streptomycin-fungizone (PSF); Life Technologies) and plated in six-well culture plates. One day following cell seeding, growth medium was replaced with serum-free medium (DMEM, 1% PSF, 1% insulin-transferrin-selenium (ITS); Life Technologies) with or without additional supplementation. There were six medium conditions: (1) serum-free control; (2) 10 ng/mL TGF-β3 (Peprotech, Rocky Hill, NJ, USA); (3) 50 μg/mL tAP; (4) 50 μg/mL tECM; (5) 50 μg/mL cAP; and (6) 50 μg/mL cECM. Media were changed every 2 days. On days 1, 3, and 7, an MTS assay (CellTiter 96® AQ_ueous_ Non-Radioactive Cell Proliferation Assay; Promega, Madison, WI, USA) was performed according to the manufacturer’s instructions. To determine the effects of soluble ECM on gene expression, 2 × 10^4^ cells/cm^2^ were plated in six-well culture plates and cultured up to 7 days, as described above. On days 1, 3, and 7, cell lysates were collected for quantitative real-time polymerase chain reaction (qPCR; described below). As significant differences across treatment groups were only seen at day 3, expression levels were normalized against day 3 controls.

### qPCR

In 2D cultures, total cellular RNA was isolated using an RNeasy Plus Mini Kit (Qiagen, Valencia, CA, USA) and reverse transcribed into cDNA through use of the SuperScript III first-strand synthesis kit (ThermoFisher Scientific). For 3D cultures, RNA isolation was preceded by homogenization of samples in Trizol (ThermoFisher Scientific). qPCR was performed using SYBR® Green master mix (Applied Biosystems, Foster City, CA, USA) on a StepOnePlus Real-Time PCR system (Applied Biosystems). Relative expression of each target was calculated using the ΔΔC_T_ method with the arithmetic average of *GAPDH* and *18S rRNA* expression used as the endogenous reference. Target gene primer sequences are listed in Additional file 1 (Table S1).

### Bioactivity of pepsin-digested ECM as three-dimensional hydrogels

To evaluate the bioactivity of pepsin-digested ECMs as 3D hydrogels, 1.0 × 10^6^ MSCs/mL were suspended in cold, pH-neutralized hydrogels consisting of the following groups: (1) collagen type I (Control; PureCol® EZ Gel, Advanced Biomatrix, Carlsbad, CA, USA); (2) tAP; and (3) cAP (all at a final concentration of 5 mg/mL). To induce thermogelation, MSC-seeded hydrogels were incubated for 1 h at 37 °C, after which reduced-serum medium (DMEM, 2% FBS, 1% PSF) was added. Constructs were collected on day 7 for qPCR.

### Bioactivity of urea-extracted ECM in culture of MSC-seeded aligned nanofibers

Sheets of aligned nanofibers were fabricated from a solution of poly-ε-caprolactone (PCL; MW = 70–90 k; Sigma-Aldrich) prepared at 15% w/v in 1:1 (v/v) tetrahydrofuran (THF; Sigma-Aldrich):dimethylformamide (DMF; ThermoFisher Scientific) as described previously [39]. The PCL solution was loaded into 10-mL syringes and extruded through an 18-gauge blunt tip needle at 3.0 mL/h using a syringe pump (PY2 70,2209, Harvard Apparatus, Holliston, MA, USA). The needle tip was placed 10 cm from a custom-designed cylindrical mandrel, which rotated at a surface velocity of 10 m/s. A DC potential of 10–18 kV (Gamma High Voltage, Ormond Beach, FL, USA) was applied to the polymer solution while an 8 kV potential was applied to two aluminum shields placed perpendicular to the mandrel axis but parallel to the needle axis, narrowing the width of the aligned nanofibrous sheet collected on the grounded mandrel.

MSCs were seeded on PCL nanofibers at 6 × 10^4^ cells/cm^2^ and cultured for 14 days in serum-reduced (2% FBS) culture medium supplemented with 50 μg/mL ascorbate-2-phosphate (Sigma-Aldrich). There were four medium conditions: (1) control; (2) 10 ng/mL TGF-β3; (3) 50 μg/mL tECM; and (4) 50 μg/mL cECM (with medium changes every 2 days). On day 14, constructs were collected for qPCR or immunofluorescence staining. qPCR was performed as described above. For immunofluorescence straining, constructs were fixed with 4% paraformaldehyde, and blocked with 1% bovine serum albumin (BSA) and 22.5 mg/mL glycine in PBS-T. Constructs were incubated with goat anti-tenomodulin (Tnmd; 1:50, sc49325 Santa Cruz Biotechnologies, Dallas, TX, USA) overnight at 4 °C. AlexaFluor 488 chicken anti-goat (Invitrogen, ThermoFisher Scientific) at a 1:500 dilution was used as the secondary antibody. Constructs were nuclear-counterstained with 4’,6-diamidino-2-phenylindole, dilactate (DAPI; Invitrogen) and imaged using a confocal microscope (Olympus FluoView 1000).

### Bioactivity of urea-extracted ECM in culture of MSC pellets

MSCs (2.5 × 10^5^/mL in 200 μL chondrogenic medium: DMEM, 1% PSF, 1% ITS, 0.1 μM dexamethasone, 40 μg/mL proline, 50 μg/mL ascorbate-2-phopshate) were distributed into conical 96-well plates and centrifuged for 10 min at 300 g. Pellets were cultured for 21 days in one of four medium conditions: (1) control; (2) 10 ng/mL TGF-β3; (3) 50 μg/mL tECM; and (4) 50 μg/mL cECM (with medium changes every 2 days). At day 21, pellets were collected for qPCR, histology, and biochemical analysis.

### Effect of TGF-β inhibition on bioactivity of urea-extracted ECM

MSC pellets were cultured for up to 21 days under one of four conditions, as described below. The small molecule TGF-β inhibitor SB-431542 (Sigma) [40] was added at a final concentration of 10 μM approximately 1 h prior to adding the appropriate culture supplement (i.e., 10 ng/mL TGF-β3, 50 μg/mL tECM, 50 μg/mL cECM). Media were changed every 2 days. qPCR, histology, and analysis of biochemical composition were performed on day 21. As supplementation with SB-431542 did not dramatically affect gene expression patterns compared to pellets cultured in control medium (i.e., without SB-431542), relative fold changes are shown normalized against the control + SB-431542 medium condition for clarity.

### Bioactivity of cartilage ECM in MSC-seeded GelMA hydrogels

GelMA was synthesized by adapting a previously established protocol [8]. Briefly, 15 g of gelatin (Type A, from porcine skin; Sigma-Aldrich) was dissolved in 500 mL deionized H_2_O at 40 °C, and then 15 mL of methacrylic anhydride (Sigma-Aldrich) was added dropwise under vigorous stirring. The mixture was placed at 37 °C in an orbital shaker at 150 rpm for 24 h. The resulting GelMA was dialyzed for 4 days against H_2_O at room temperature using 2000 NMWCO dialysis tubing (Sigma-Aldrich) to completely remove all low-molecular-weight byproducts, with changes in H_2_O twice daily. After lyophilization, the product was stored at −20 °C until future use. Prior to use, GelMA was reconstituted at 10% (w/v) in HBSS. The visible light-sensitive photoinitiator lithium phenyl-2,4,6-trimethylbenzoylphosphinate (LAP) was then added at 0.25% v/v and dissolved by gentle shaking at room temperature. MSCs were homogeneously suspended in one of two hydrogels: (1) 10% w/v GelMA (control); or (2) 10% w/v GelMA supplemented with 500 μg/mL cECM (cECM) at a concentration of 20 × 10^6^ cells/mL. Before gelation, MSC-seeded hydrogels (~50 μL) were distributed to cylindrical silicone molds measuring 5-mm diameter × 2-mm depth. To induce photogelation, hydrogels were exposed to 2 min of visible light (450–490 nm). MSC-seeded hydrogels were then removed from the silicone molds and transferred to six-well plates previously coated with silicone (Sigmacote, Sigma-Aldrich) to prevent cell migration and adhesion onto the plastic surface. Constructs were cultured up to 21 days in chondrogenic medium with or without an additional 10 ng/mL TGF-β3 (Peprotech) supplementation. Medium was changed every 2 days. Constructs were collected on days 7 and 21 for analysis of gene expression and biochemical content.

### Histology and immunofluorescence

All samples collected for histology (excluding electrospun nanofibers) were fixed in 10% phosphate-buffered formalin, serially dehydrated, embedded in paraffin, and sectioned (6 μm thickness) with a microtome (Leica RM2255, Leica Biosystems, Buffalo Grove, IL, USA). Samples were rehydrated and stained with hematoxylin and eosin (H&E; Sigma-Aldrich), Safranin O and Fast Green (Electron Microscopy Sciences, Hatfield, PA, USA), or DAPI (Life Technologies, Carlsbad, CA, USA).

For samples used for immunofluorescence staining, antigen retrieval entailed incubation with chondroitinase ABC (100 mU/mL) and hyaluronidase (250 U/ml) suspended in 0.02% BSA for 30 min at 37 °C. Samples were incubated overnight at 4 °C with the following primary antibodies: 1:400 rabbit anti-collagen type II (ab34712, Abcam, Cambridge, MA, USA), 1:400 mouse anti-collagen type I (5D8-G9/Col1, ThermoScientific), or 1:400 mouse anti-collagen type X (ab49945, Abcam). Samples were incubated in one of two secondary antibodies for 1 h at room temperature: 1:500 AlexaFluor 488 goat anti-mouse or AlexaFluor 594 goat anti-rabbit (Invitrogen, ThermoFisher Scientific).

Samples were photographed using an Olympus SZX16 stereomicroscope under bright field (H&E, Safranin O) or epifluorescence (excitation: 405 nm for DAPI; 488 nm (green) or 594 nm (red) for immunofluorescence.

### Biochemical composition

To determine the biochemical composition of tissues and 3D constructs, dried samples were digested overnight at 65 °C at a concentration of 10 mg/mL in a digestion buffer (pH 6.0) containing 2% papain (v/v, from Papaya latex; Sigma-Aldrich), 0.1 M sodium acetate, 0.01 M cysteine HCl, and 0.05 M EDTA. Concentrated NaOH was subsequently added to the digestion solution to adjust the pH to 7.0. Sulfated glycosaminoglycan (sGAG) content was quantified with a Blyscan Assay according to the manufacturer’s instructions (Biocolor, Carrickfergus, UK). dsDNA content was determined using the Quant-iT Picogreen dsDNA assay (Invitrogen). Total collagen content was determined using a modified hydroxyproline assay. Briefly, 200 μL of each sample was hydrolyzed with an equal volume of 4 N NaOH at 121 °C for 75 min, neutralized with an equal volume of 4 N HCl, and then titrated to an approximate pH of 7.0. The resulting solution was combined with 1.2 mL chloramine-T (14.1 g/L) in buffer (50 g/L citric acid, 120 g/L sodium acetate trihydrate, 34 g/L NaOH, and 12.5 g/L acetic acid) and allowed to stand at room temperature for 30 min. The solution was then combined with 1.2 mL of 1.17 mM p-dimethylaminobenzaldehyde in perchloric acid and placed in a 65 °C water bath for 20 min; 200 μL of each sample was added to a clear 96-well plate, in duplicate, and absorbance at 550 nm was read. PureCol bovine collagen (3.2 mg/mL; Advanced Biomatrix) was serially diluted to provide a standard curve ranging from 0 to 1000 μg/mL.

### Statistics

Comparisons across multiple conditions or time points were made using a one-way or two-way analysis of variance (ANOVA) with Tukey post-hoc testing for multiple comparisons. When comparing two conditions, a Student’s *t* test was performed. Data are presented as mean ± standard deviation. Experiments were performed with biological triplicates over at least three independent trials. Sample sizes are indicated in figure legends. Statistical significance was considered at *p* < 0.05.

## Results

### Characterization of soluble tendon- and cartilage-derived extracellular matrix

**Fig. 1:**
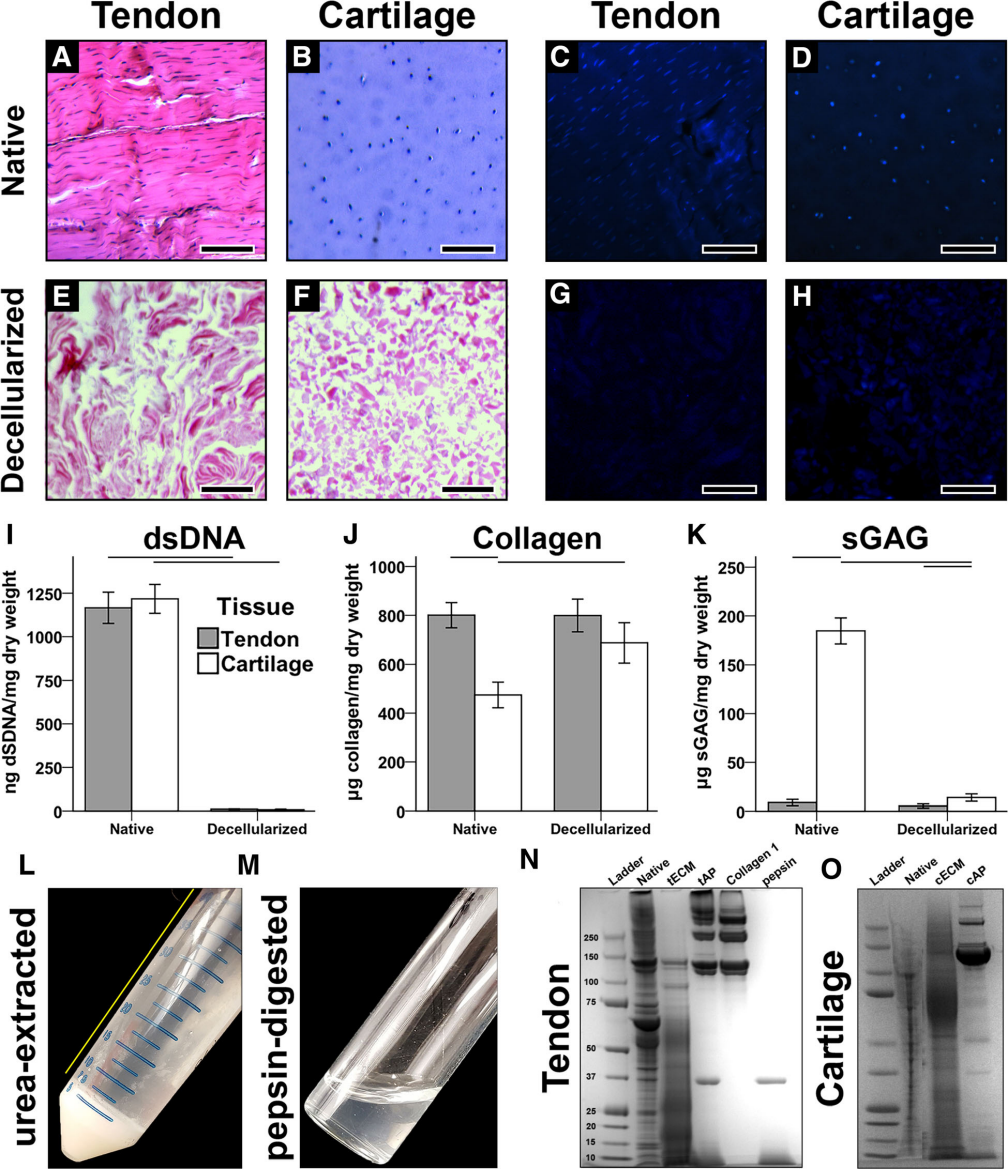
Characterization of soluble tendon- and cartilage-derived extracellular matrix. **a**–**d** Prior to decellularization, nuclei are clearly present in native tendon and cartilage tissues, as shown through H&E and DAPI staining. **e**–**h** Following decellularization, no nuclei are visible. *Scale bar* = 200 μm. **i** dsDNA contents were significantly reduced in decellularized tissues compared to native tissues (*p* < 0.001, *n* = 8). **j** Collagen content in native and decellularized tendon was equivalent, but was increased in decellularized cartilage vs. native cartilage (*p* < 0.05, *n* = 8). **k** Sulfated glycosaminoglycan (*sGAG*) content was higher in cartilage tissues than tendon tissues, regardless of decellularization step (*p* < 0.05), but decellularized cartilage contained significantly less sGAG than native cartilage (*p* < 0.001, *n* = 8). For (**i**–**k**), statistically significant differences are indicated by overlying horizontal lines. **l** Urea extraction yielded an insoluble and soluble fraction. The soluble supernatant (*yellow line*) was collected. **m** Pepsin digestion yielded a homogeneous slurry. **n**,**o** SDS-PAGE analysis of tendon (**n**) and cartilage (**o**) tissues at different stages of decellularization and solubilization. *cAP* acid-pepsin digested cartilage extracellular matrix, *cECM* urea-extracted cartilage extracellular matrix, *tAP* acid-pepsin digested tendon extracellular matrix, *tECM* urea-extracted tendon extracellular matrix

The decellularization protocol successfully reduced cellular content from both tendon and cartilage, as evidenced by the absence of nuclei on both H&E- and DAPI-stained sections (Fig. 1a–h), as well as a significant reduction in dsDNA content (Fig. 1i). Per dry weight, the total collagen contents of native and decellularized tendon were equivalent, while decellularized cartilage exhibited a significant increase in collagen content with a corresponding loss in sGAG content (Fig. 1j–k). The majority of decellularized tissue powder was insoluble in urea (Fig. 1l) but was homogenously digested by the acid-pepsin solution (Fig. 1m). As a result, as shown in Fig. 1n and o, SDS-PAGE showed that urea-extracted tendon ECM (tECM) and cartilage ECM (cECM) were enriched for low to moderate molecular weight proteins, with only faint bands corresponding to collagen α chains. Conversely, the pepsin-digested tendon (tAP) and cartilage (cAP) were principally composed of collagen types I and II, respectively, with very faint bands found in the low to moderate molecular weight regions. The prominent streak in the well of native cartilage is an artifact attributable to the high proteoglycan content (Fig. 1o). tECM and cECM possessed a higher growth factor content than their pepsin-digested counterparts, with notable differences in basic fibroblast growth factor (bFGF) and TGF-β1 (Additional file 2: Table S2).

### Effect of soluble ECM on human MSCs in 2D culture

**Fig. 2:**
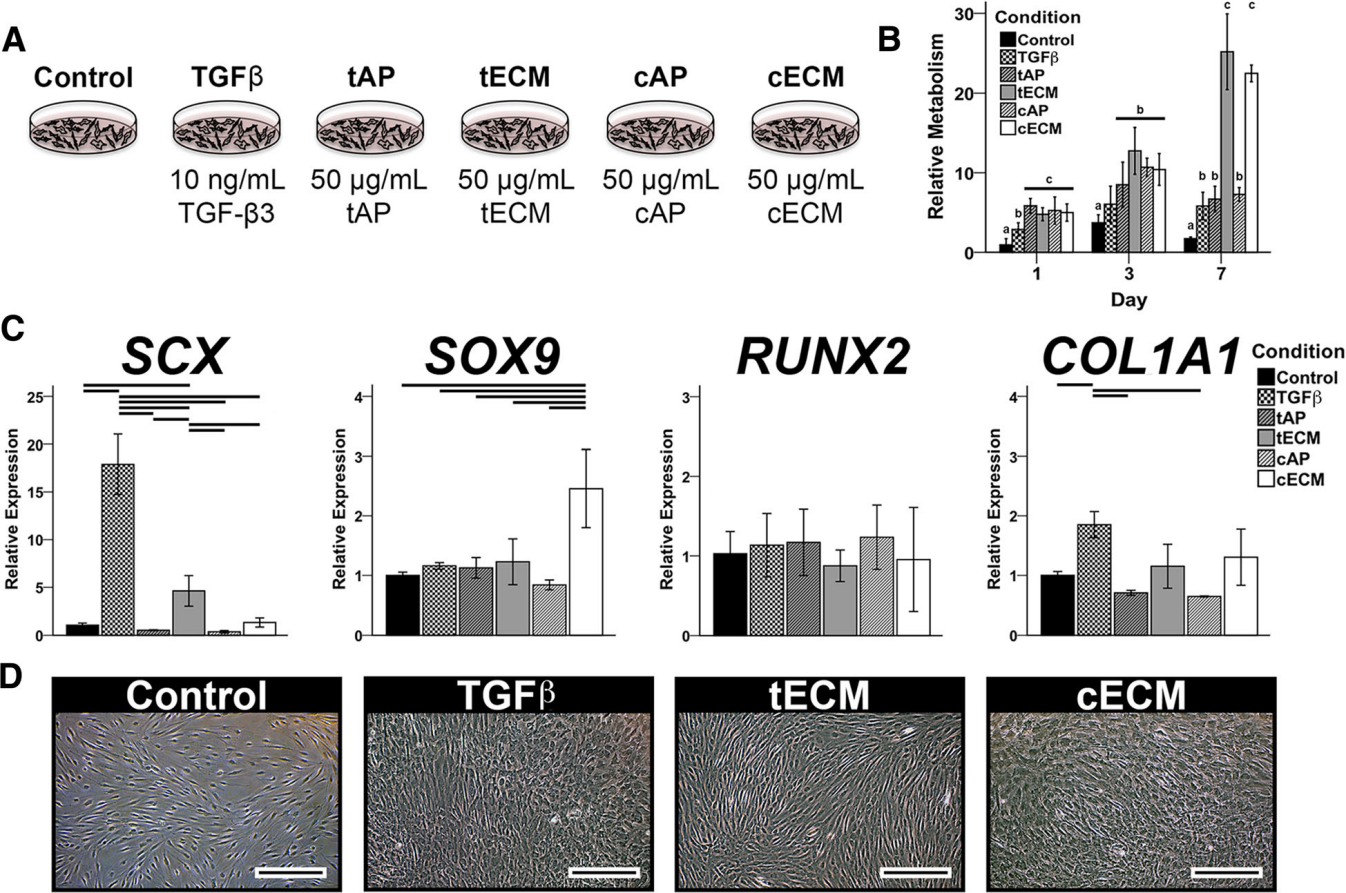
Effect of soluble ECMs on human MSCs in 2D culture. **a** MSCs were cultured up to 7 days on tissue culture plastic in one of six medium conditions. **b** MTS assay showed that all ECM groups enhanced cell metabolism (proliferation), but urea-extracted fractions were the most mitogenic by day 7. Letters correspond to statistically equivalent groups at a given timepoint. **c** Gene expression analysis on day 3 suggested tissue-specific bioactivity of urea-extracted ECM fractions (*p* < 0.05, *n* = 9). Statistically significant differences are indicated by overlying *horizontal lines*. **d** Phase contrast microscopy showed spindle-shaped cells in the tECM group but cobblestone morphology with TGF-β and cECM supplementation. *Scale bar* = 100 μm. *cAP* acid-pepsin digested cartilage extracellular matrix, *cECM* urea-extracted cartilage extracellular matrix, *tAP* acid-pepsin digested tendon extracellular matrix, *tECM* urea-extracted tendon extracellular matrix, *TGF* transforming growth factor

Human MSCs were grown as 2D cultures seeded on plastic in one of six medium conditions (Fig. 2a). Pepsin-digested and urea-extracted ECM supplementation enhanced cell proliferation, with the urea-extracted groups showing the greatest effect by day 7 (Fig. 2b). Only the urea-extracted ECMs upregulated tissue-specific transcription factors; tECM preferentially enhanced *SCX* expression while cECM upregulated *SOX9* expression (Fig. 2c). No soluble ECM preparation affected expression of the osteogenic marker *RUNX2*. Collagen type II (*COL2A1*) and aggrecan (*ACAN*) expression was not detectable, while collagen type I (*COL1A1*) was only significantly upregulated by TGF-β3, which also greatly increased *SCX* expression (Fig. 2c). MSCs grown in tECM-supplemented medium possessed a spindle-shaped morphology, while cECM and, to a lesser extent, TGF-β3 supplementation produced a cobblestone morphology (Fig. 2d).

### Effect of soluble ECM on MSCs seeded on aligned nanofibers

**Fig. 3:**
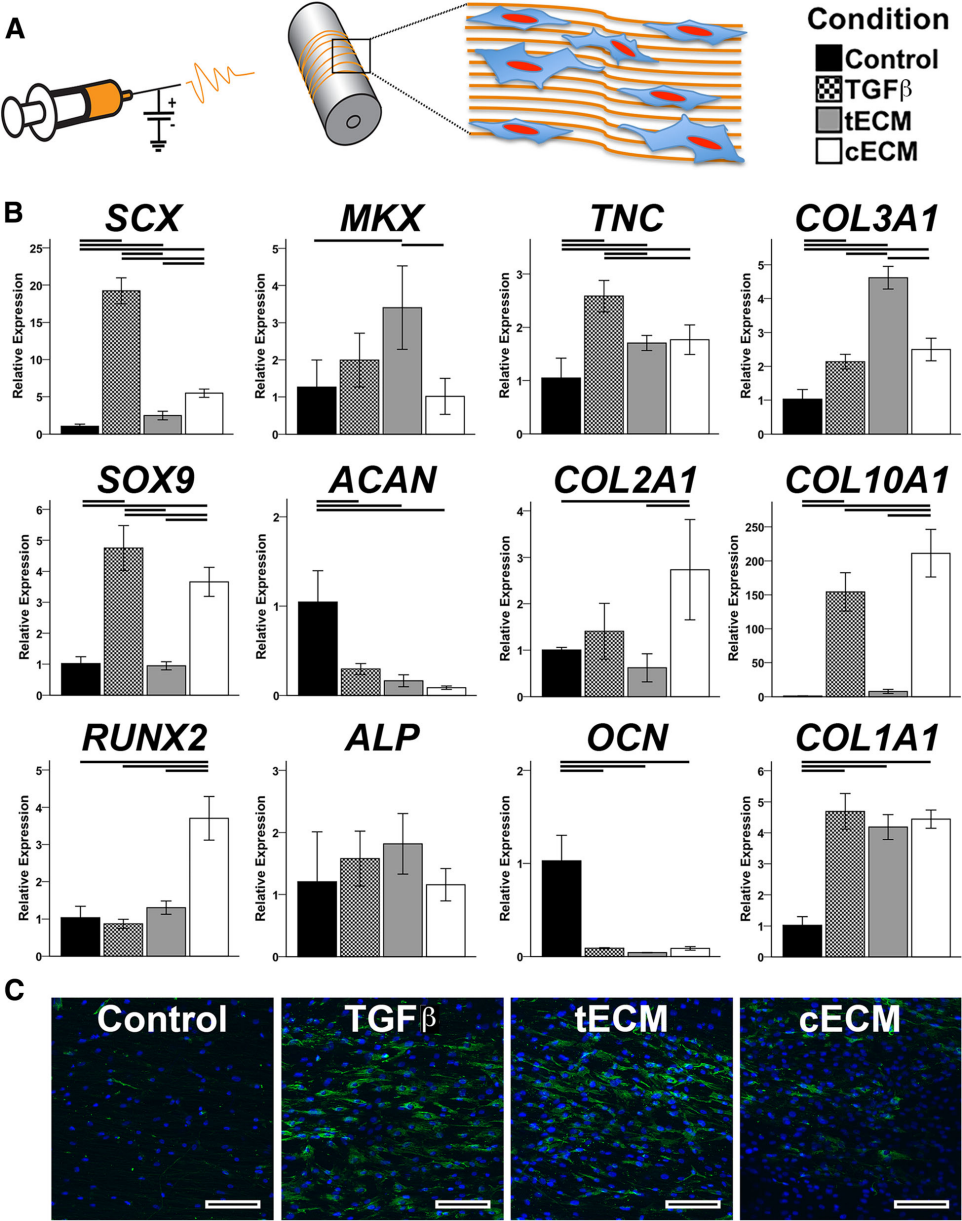
Effect of soluble ECM on MSCs seeded on aligned nanofibers. **a** MSCs were cultured on aligned PCL nanofibers in one of four medium conditions. **b** Gene expression analysis on day 14 showed tenogenic and chondrogenic effects due to TGF-β supplementation. tECM supplementation promoted a tenogenic phenotype while cECM upregulated chondrogenic markers and *RUNX2*. Both TGF-β and cECM increased expression of the hypertrophic marker *COL10A1* (*p* <0.05, *n* = 9). Statistically significant differences are indicated by overlying *horizontal lines*. **c** Immunofluorescence staining of tenomodulin shows increasing intensity in the following order: control < cECM < tECM < TGF-β; Tnmd = *green*, nuclei = *blue. Scale bar* = 100 μm. *cECM* urea-extracted cartilage extracellular matrix, *tECM* urea-extracted tendon extracellular matrix

MSCs seeded on aligned PCL nanofibers became elongated in the direction of the fibers (data not shown). TGF-β3 supplementation upregulated both tenogenic (*SCX*, *TNC*, *COL3A1*, *COL1A1*) and chondrogenic (*SOX9*, *COL10A1*) markers, while tECM supplementation enhanced expression of tenogenic markers only (Fig. 3b). cECM modestly increased tenogenic markers (*SCX*, *TNC*, *COL3A1*) but upregulated chondrogenic markers (*SOX9, COL2A1, COL10A1*) to an equivalent or greater extent than TGF-β3; cECM also upregulated *RUNX2*. All supplements decreased gene expression of the cartilage proteoglycan *ACAN* and bone protein osteocalcin (*OCN*). Paralleling the expression pattern of *SCX*, an upstream driver of tenomodulin (*TNMD*) [41], confocal microscopy revealed the greatest immunostaining intensity for Tnmd in the TGF-β3 group. However, tECM enhanced Tnmd production to a greater extent than cECM (Fig. 3c).

### Effect of soluble ECM on MSC pellets

**Fig. 4:**
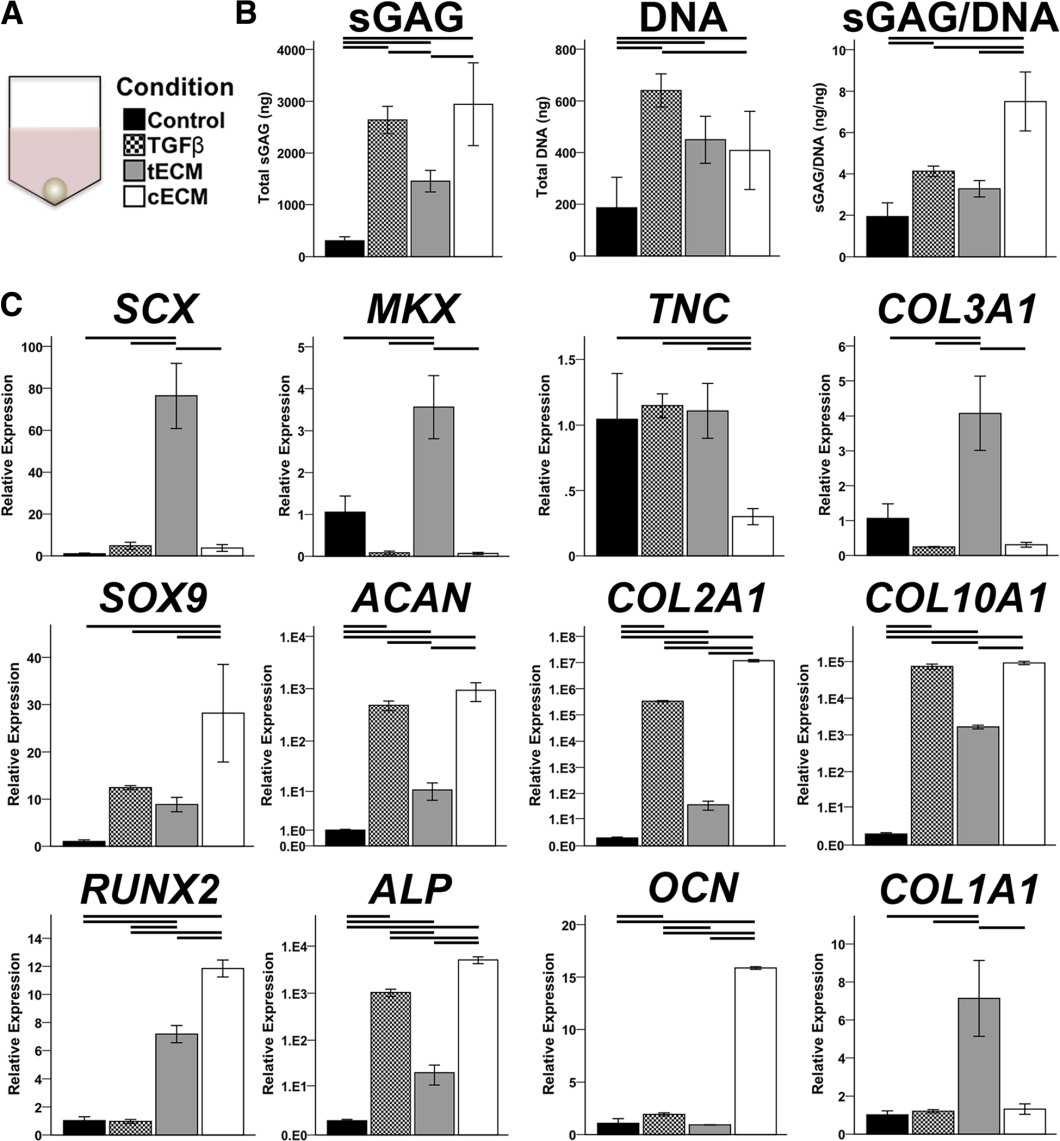
Effect of soluble ECMs on MSC pellet biochemical composition and gene expression. **a** MSC pellets were cultured in one of four medium conditions for 21 days. **b** Biochemical composition of pellets reveals anabolic and mitogenic effects for all supplements; cECM promoted the greatest relative sGAG production (p < 0.05, n = 9). **c** Gene expression analysis on day 21 shows chondrogenic effects of TGF-β, tenogenic effects of tECM, and chondrogenic and osteogenic effects of cECM (p < 0.05, n = 9). Statistically significant differences are indicated by overlying *horizontal lines. cECM* urea-extracted cartilage extracellular matrix, *sGAG* sulfated glycosaminoglycan, *tECM* urea-extracted tendon extracellular matrix

**Fig. 5:**
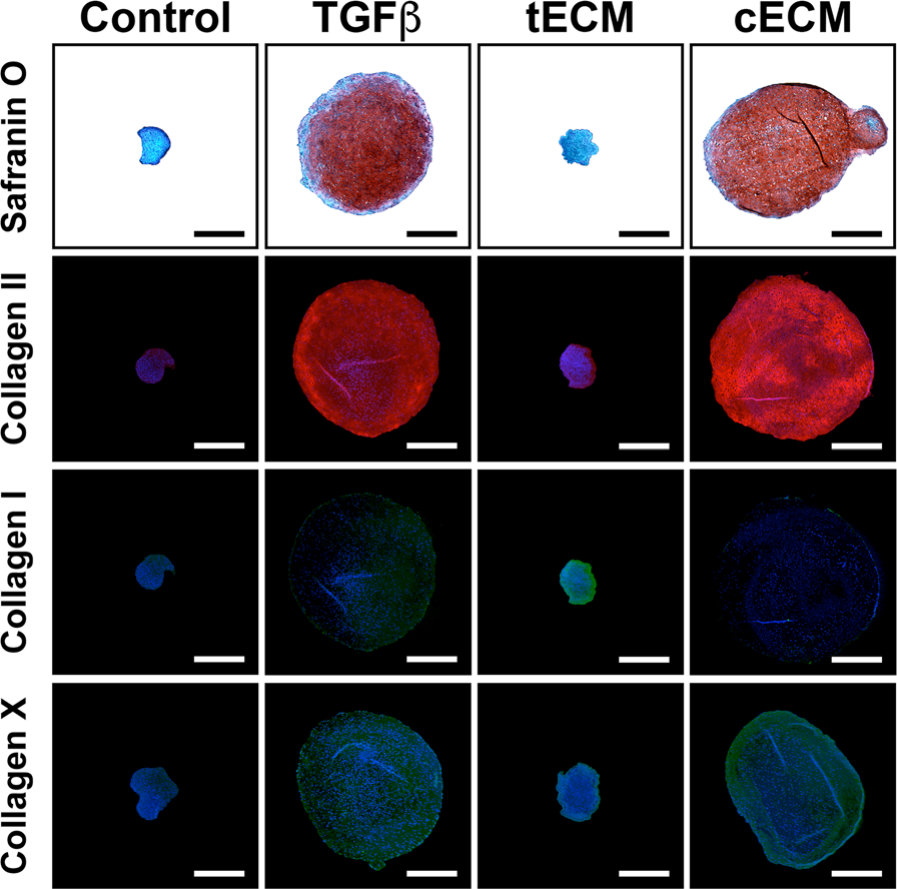
Effect of treatment with soluble ECMs on production of specific ECM components in MSC pellet cultures. Supplementation with TGF-β and cECM promoted deposition of sGAG, Collagen II, and Collagen X, while tECM enhanced Collagen I synthesis. Control consisted of MSC pellet culture without any medium supplement. Collagen II = *red*, Collagen I = *green*, Collagen X = *green*, nuclei = *blue. Scale bar* = 500 μm. *cECM* urea-extracted cartilage extracellular matrix, *tECM* urea-extracted tendon extracellular matrix, *TGF* transforming growth factor

Total and relative sGAG contents were increased in pellets cultured in supplemented medium (Fig. 4b). TGF-β3 and cECM supplementation increased total sGAG to a similar extent, but cECM was superior when sGAG content was normalized to dsDNA content (Fig. 4b). In terms of gene expression (Fig. 4c), TGF-β3 preferentially promoted a chondrogenic phenotype as shown by increased expression of *SOX9*, *ACAN*, and *COL2A1*. tECM promoted a tenogenic phenotype with robust upregulation of *SCX*, *MKX*, *COL3A1*, and *COL1A1*, with more modest effects on chondrogenic and osteogenic markers. Similarly, cECM had a negligible or inhibitory effect on tenogenic markers but promoted chondrogenesis to an equivalent or greater degree than TGF-β3. However, cECM also upregulated osteogenic markers most strongly, as seen in expression patterns of *COL10A1* (hypertrophic marker), *RUNX2*, *ALP*, and *OCN* (Fig. 4c). Histological analysis of pellets showed a pattern that was consistent with assays for biochemical composition and gene expression. Namely, TGF-β3 and cECM enhanced proteoglycan and collagen II deposition while tECM pellets showed the greatest collagen I staining intensity (Fig. 5).

### Effect of TGF-β inhibition on bioactivity of soluble ECM

**Fig. 6:**
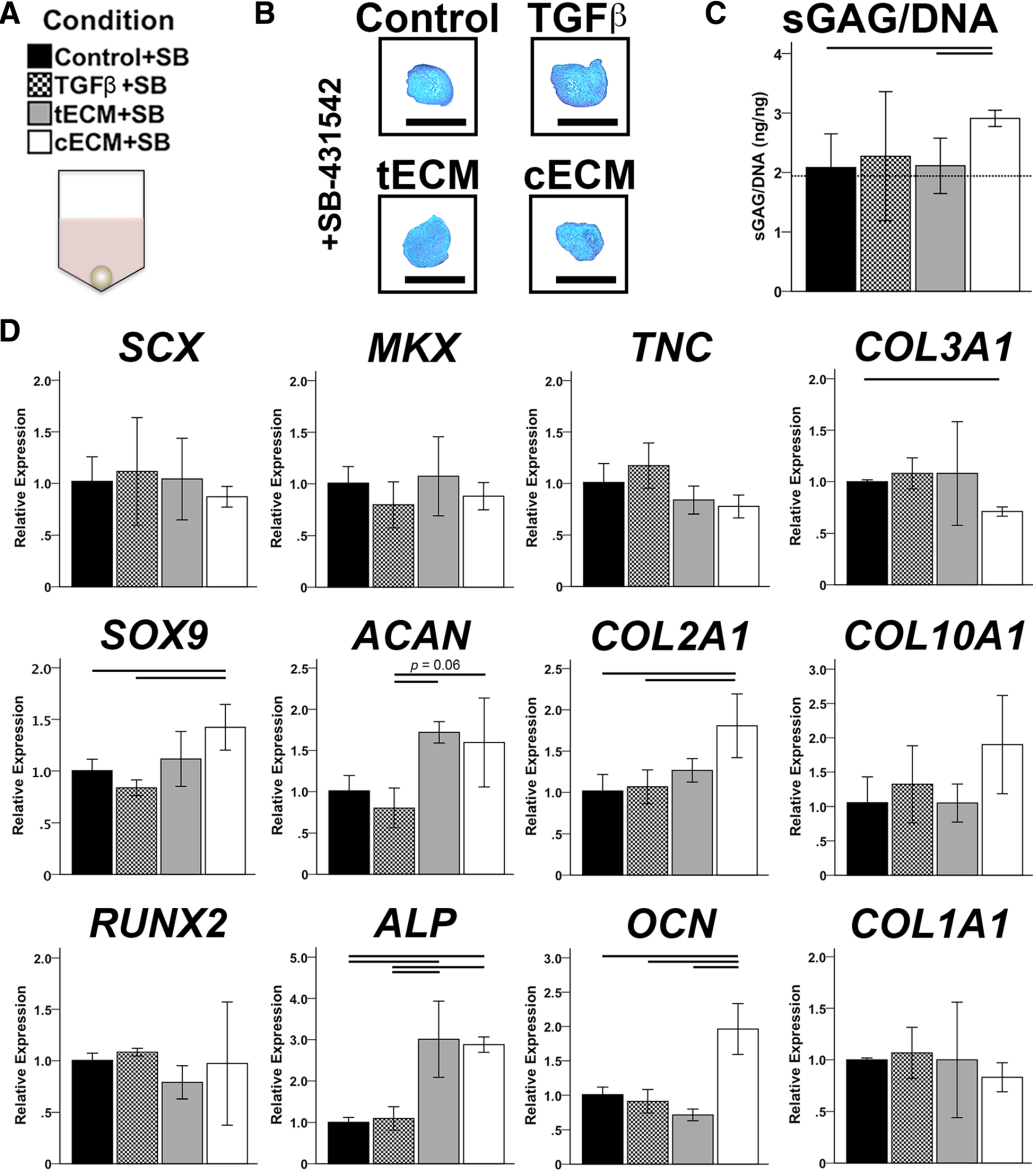
Effect of TGF-β inhibition on bioactivity of soluble ECM. **a** Medium conditions for pellet cultures were further supplemented with 10 μM SB-431542. **b** Safranin O staining. *Scale bar* = 500 μm. **c** Normalized sGAG contents show blunted anabolic effects of medium supplements (*p* < 0.05, *n* = 9); *dotted line* indicates sGAG/dsDNA content of control medium (without SB-431542). **d** Gene expression analysis shows complete inhibition of exogenous TGF-β and blunted tissue-specific bioactivity of ECM supplements (*p* < 0.05, *n* = 9). In (**c** and **d**), statistically significant differences are indicated by overlying *horizontal lines. cECM* urea-extracted cartilage extracellular matrix, *SB* SB-431542, *sGAG* sulfated glycosaminoglycan, *tECM* urea-extracted tendon extracellular matrix, *TGF* transforming growth factor

The activity of endogenous TGF-β in tECM and cECM was blocked using SB-431542, a TGF-β receptor type 1 antagonist [40] that was added at 10 μM to pellet culture media (Fig. 6a). Treatment with SB-431542 significantly reduced the anabolic effects of TGF-β and cECM on pellets, as evidenced by the loss of proteoglycan staining (Fig. 6b) and sGAG content (Fig. 6c; Additional file 3: Figure S1). Gene expression analysis showed that SB-431542 treatment eliminated the tenogenic effect of tECM and the chondrogenic effect of TGF-β (Fig. 6d). Interestingly, cECM supplementation still promoted significant increases in *SOX9*, *ACAN*, and *COL2A1* expression despite treatment with SB-431542 (Fig. 6d), although these increases were far weaker than pellets treated with cECM in the absence of SB-431542 (Fig. 4c). ECM-mediated upregulation of osteogenic markers, alkaline phosphatase (*ALP*) and *OCN*, also persisted in the presence of TGF-β inhibition.

### Independent and synergistic effects of cECM and TGF-β on chondrogenesis of MSCs seeded in 3D photocrosslinked GelMA hydrogels

**Fig. 7:**
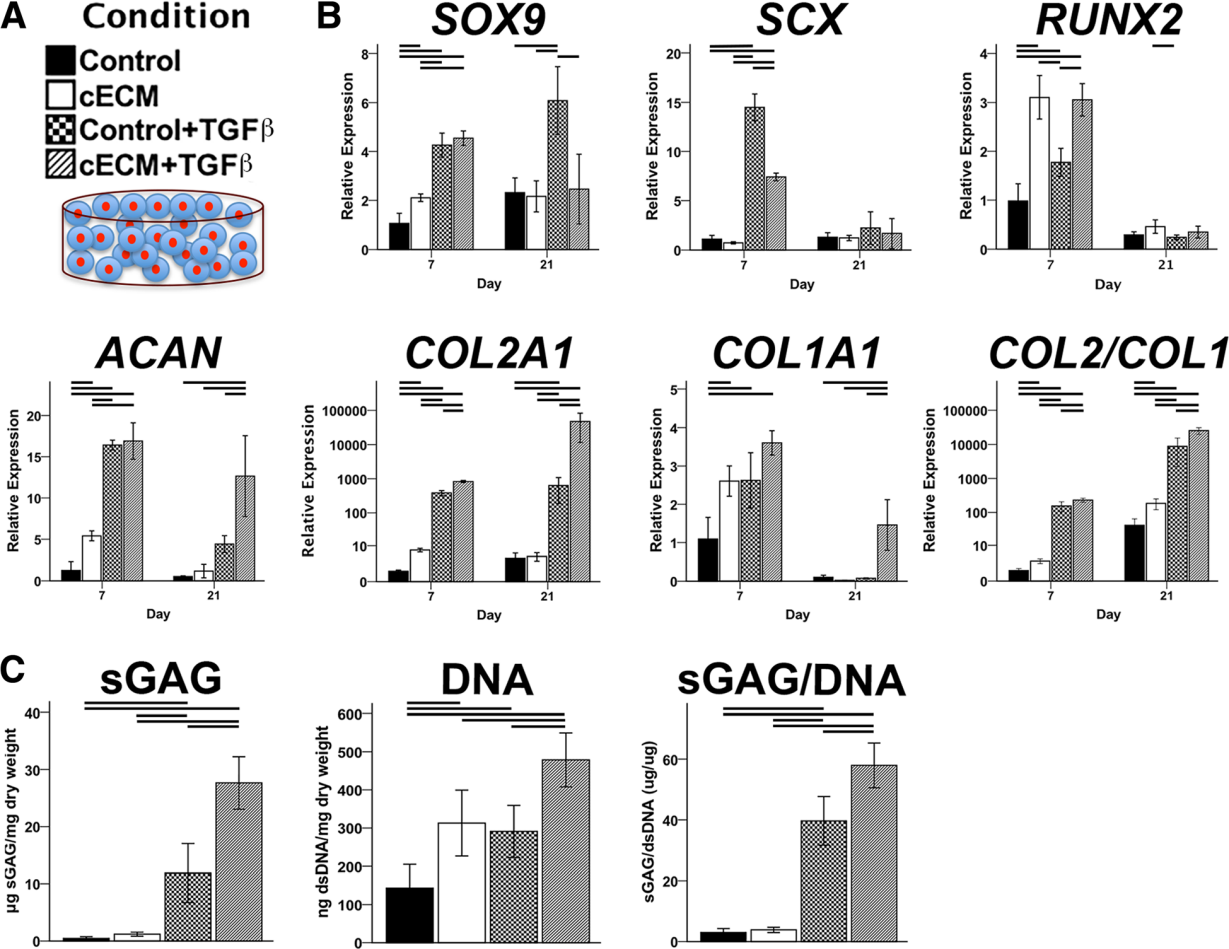
Independent and synergistic effects of cECM and TGF-β on chondrogenesis of MSCs seeded in 3D photocrosslinked GelMA hydrogels. **a** MSC-seeded GelMA hydrogel constructs, with or without cECM enhancement, were cultured in chondrogenic medium, with or without TGF-β supplementation, for up to 21 days. **b** Gene expression analysis shows independent and synergistic effects of cECM and TGF-β (*p* < 0.05, *n* = 9). **c** Biochemical composition analysis shows synergistic effect of cECM and TGF-β in enhancing absolute and normalized sGAG production (*p* < 0.05, *n* = 9). Statistically significant differences are indicated by overlying *horizontal lines. cECM* urea-extracted cartilage extracellular matrix, *sGAG* sulfated glycosaminoglycan, *TGF* transforming growth factor

MSCs were seeded in photocrosslinked GelMA hydrogels and cultured in chondrogenic medium (with or without TGF-β supplementation) for up to 21 days (Fig. 7a). On day 7, the inclusion of cECM within the hydrogels had independently upregulated chondrogenic markers *SOX9*, *ACAN*, and *COL2A1*, as well as the ratio of *COL2A1:COL1A1*, despite a modest increase in *COL1A1* (Fig. 7b). *RUNX2* expression was also upregulated by cECM on day 7, but was equivalent to controls (and returned to baseline) by day 21. Supplementation of culture medium with TGF-β dramatically enhanced the expression of chondrogenic markers, compared to controls, on days 7 and 21. The effect was further enhanced when cECM was mixed with the GelMA hydrogel, suggesting a synergistic effect between the cECM and TGF-β (Fig. 7b). This synergistic effect was also confirmed upon analysis of the biochemical composition of MSC-seeded hydrogels (Fig. 7c). Importantly, a cellular cECM-containing GelMA hydrogels had negligible sGAG content (data not shown), suggesting that the observed group differences are attributable to the effects of cECM on MSCs rather than sGAG contained within cECM solution.

## Discussion

Given the conservation of ECM proteins across species, the utility of decellularized tissues as biomaterials capable of promoting tissue-specific cell phenotypes is theoretically and empirically supported [42, 43]. Nevertheless, there is an inherent trade-off between tissue processing and retention of bioactive cues [16]. While sufficient decellularization is required to mitigate an adverse immune response to the implanted scaffold [44], it is uncertain which elements of the ECM must be preserved in order to retain homologous bioactivity. Pepsin digestion of decellularized ECM yields viscous slurries capable of undergoing thermoresponsive gelation when pH balanced [38, 45], providing an attractive biomaterial for minimally invasive cell delivery to irregularly shaped defects. On the other hand, characterization of pepsin-digested ECM is seldom performed.

Our group [35] and others [32, 46] have recently reported that pepsin solubilization produces digests composed principally of structural ECM proteins, especially collagen; it should be pointed out that acid-pepsin digestion is the longstanding, classic method of tissue collagen isolation [47]. A similar finding was seen in this study. Notably, small to moderate molecular weight proteins were abundantly found in urea-extracted ECM while the respective collagen α chains constituted the majority of protein found in pepsin-digested ECM solutions. These compositional differences were demonstrated both qualitatively through SDS-PAGE (Fig. 1n and o) and quantitatively through a growth factor array (Additional file 2: Table S2). The compositional differences resulting from solubilization protocols presumably underlie the observed differences in bioactivity mediated by the ECM when delivered as medium supplements to MSCs cultured on 2D plastic. However, it may be argued that higher concentrations of pepsin-digested ECMs would be required to induce similar effects as urea-extracted ECMs, given the relative predominance of collagens (at the expense of small to moderate molecular weight proteins) in the former. To that end, supplementation of medium with a ten-fold higher concentration of pepsin-digested ECMs (i.e., 500 μg/mL) did not alter their effects of MSC proliferation or differentiation (data not shown). Similarly, when MSCs were seeded in 3D thermoresponsive hydrogels derived from pepsin-digested ECMs, tissue-specific bioactivity did differ between groups (Additional file 4: Figure S2). While limited, the literature corroborates these findings. Pati et al. [32] reported only a ~1.5-fold increase in *SOX9* and *COL2A1* expression when human MSCs were seeded in cAP hydrogels, as compared to collagen I hydrogels. Likewise, two related studies [29, 48] found a negligible effect of pepsin-digested cartilage ECM, compared to controls, in enhancing chondrogenesis.

In contrast, we previously found that treatment with tECM, a preparation of urea-extracted tendon ECM, upregulated expression of tenogenic markers, with concurrent downregulation of osteogenic markers, in MSCs cultured in a hydrogel under static uniaxial tension [37]. Zhang et al. [36] reported similar tissue-specific findings when coating tissue culture dishes with urea-extracted ECM derived from skin, skeletal muscle, and liver. Building on these findings, this study showed that urea-extracted tECM and cECM upregulated tissue homologous transcription factors (i.e., *SCX* and *SOX9*, respectively) in MSCs grown on 2D plastic, while exogenous TGF-β3 preferentially upregulated *SCX* alone. However, these effects were not sustained beyond 7 days, likely attributable to the stress of sustained serum-starvation coupled with the nonphysiologic biophysical microenvironment (i.e., 2D culture on plastic).

Nevertheless, as these initial experiments supported a superiority of urea-soluble ECM compared to pepsin-digested ECM, we thereafter utilized only urea-extracted tECM and cECM to explore their tissue-specific bioactivity in two different 3D microenvironments: aligned electrospun nanofibers and cell pellets.

Electrospun nanofibers mimic the structural aspects of musculoskeletal tissue ECM (e.g., collagen fibrils) and are capable of directing cell behavior [49]. In particular, aligned nanofibers, reminiscent of the aligned collagen I fibrils of native tendon, have been shown to promote tenogenic differentiation of seeded stem cells [5, 6, 50]. Leung et al. [10] reported that supplementation with TGF-β3 further enhanced tenogenesis in MSCs seeded on aligned chitosan-PCL nanofibers, while Kishore et al. [51] found no additional effect of bone morphogenetic protein (BMP)-12 supplementation with MSCs seeded on electrochemically aligned collagen threads. Similarly, nonaligned PCL nanofibers coated with pulverized tendon ECM showed little benefit over nanofibers alone in promoting a tenogenic phenotype of seeded MSCs [52]. These conflicting results suggest a complex interaction between physical and biochemical cues in directing cell differentiation. On the other hand, in this study, tECM supplementation was found to further upregulate expression of tenogenic markers, with negligible or inhibitory effects on chondrogenic and osteogenic expression, in MSCs seeded on aligned nanofibers. In comparison, cECM affected expression of chondrogenic markers to a similar extent as TGF-β3, with a relatively diminished effect on tenogenic markers. Of note, *SCX* is known to cooperatively regulate *SOX9* and *COL2A1* expression in the context of chondrogenesis [53] despite its common categorization as a tendon-specific marker [54], perhaps explaining the small but significant upregulation of *SCX* mediated by cECM. In contrast, TGF-β3 upregulated markers of both tenogenesis and chondrogenesis. Taken together, these findings support the tissue-specific bioactivity of urea-extracted ECMs when cells are seeded on aligned nanofibers. The results parallel those of Sun et al. [55], who reported increased osteogenesis when gelatin nanofibers were enhanced with noncollagenous proteins extracted from bone using a similar method to this study.

As aligned electrospun nanofibers were used to mimic the ultrastructure of native tendon, pellet cultures were employed as an in vitro assay to replicate the early condensation, and subsequent chondrogenesis, of mesenchymal cells in limb skeletal development. In this context, TGF-β3 preferentially promoted chondrogenesis, while tECM and cECM promoted homologous gene expression (i.e., tenogenesis and chondrogenesis, respectively). TGF-β is essential for mediating chondrogenesis in vivo [13, 56], yet its in vitro effect is variable and dependent on other microenvironmental cues. For instance, Lorda-Diez et al. [57, 58] identified several downstream regulators of TGF-β signaling that mediated either fibrogenic or chondrogenic differentiation. Interestingly, in this study, inhibition of TGF-β type 1 activin receptor-like kinase receptors ALK4, ALK5, and ALK7 by SB-431542 [40] largely abolished the tissue-specific bioactivity of tECM and cECM, suggesting that TGF-β signaling is necessary, but not sufficient, to explain their tissue-specific effects.

A growth factor array analysis revealed significant differences in composition between tECM and cECM; it is possible that the greater concentrations of bFGF, BMP-5, and BMP-7 present in cECM mediated the cartilage-specific effects. However, given their role in bone formation [59], the additional BMPs found in cECM may also have contributed to the noted upregulation of hypertrophic and osteogenic markers seen in this study, although TGF-β alone also induced some degree of hypertrophy in pellet cultures. The finding of cartilage ECM-induced hypertrophy has also been recently reported in similar studies [60, 61]. Indeed, the stability of the chondrogenic phenotype remains a persistent challenge in cartilage tissue engineering [15]. It is possible that the use of cECM derived from adult animals, either alone or in combination with other prochondrogenic factors, could promote chondrogenic differentiation with less hypertrophy, as the delineation between articular cartilage and subchondral bone (with obvious vasculature) is apparent (Additional file 5: Figure S3), allowing for the isolation of cartilage ECM alone. As shown in the growth factor array data (Additional file 2: Table S2), cECM from 2- to 3-year-old animals contains a lower concentration of growth factors than cECM from 6- to 8-week-old animals (as used in this study), but also greatly reduced levels of BMPs. Clearly, further elucidation of the composition of the soluble ECM preparations, and the interactions among these elements, will be necessary to expand on the findings obtained herein.

We propose that inclusion of urea-extracted ECM may be considered as a component of engineered scaffolds to accelerate or enhance the differentiation of encapsulated MSCs towards a particular tissue-specific lineage. The inclusion of cartilage ECM structural proteins such as collagen type VI [62], collagen type II, and proteoglycans [63, 64] within MSC-seeded hydrogels has been found to enhance chondrogenic differentiation. Similarly, Almeida et al. [28, 65] reported similar improvements when using decellularized cartilage ECM particles. In this study, cECM was mixed with photocrosslinkable GelMA hydrogels, a biomaterial which we previously found to support robust chondrogenesis [8]. cECM independently enhanced chondrogenesis at day 7, but the chondroinductive effect decreased by day 21. Rather, medium supplementation with TGF-β was required for sustained upregulation of chondrogenic markers, with corresponding deposition of cartilage ECM proteins; this finding agrees with related work [27, 28, 65]. Despite the apparent necessity of exogenous TGF-β for robust cartilage formation, cECM within the hydrogel interacted synergistically with the supplemented TGF-β, as demonstrated by the greatest increases in chondrogenic gene expression and sGAG deposition seen in the cECM + TGF-β group. Given these results, we are now developing cECM-enhanced hydrogels with controlled release of encapsulated TGF-β, potentially obviating the need for medium supplementation and improving the translational applicability of this approach.

Although this study found that urea-extracted ECM, rather than pepsin-digested ECM, is capable of promoting tissue-specific cell phenotypes across multiple culture conditions, we did not explore the many other benefits reported for pepsin-solubilized ECM. In particular, pepsin ECM digests have been found to enhance in vitro cell migration [34], proliferation [66], and macrophage polarization [67], effects influenced by tissue source [68], animal age [69], and fraction [70]. In vivo, ECM-mediated effects on macrophage polarization, and the broader inflammatory response, at least partially explain the benefit of ECMs in promoting constructive remodeling (i.e., improved healing) [71, 72]. To what extent in vitro assays for tissue-specific differentiation are predictive of enhanced in vivo healing remains unknown. For instance, Keane et al. [34] and Wolf et al. [45] found that ECM hydrogels derived from esophagus and skeletal muscle, respectively, promoted tissue-specific differentiation of cells in vitro, but their effects in vivo were not superior to ECM hydrogels derived from heterologous tissues. The effects of urea-extracted ECM fractions on macrophage polarization and in vivo healing were beyond the scope of the present investigation, but certainly are worthy of future inquiry. Indeed, it is well recognized that successful regeneration of musculoskeletal tissues will require a greater understanding of the intersection of biomimetic biomaterials that are capable of guiding tissue-specific cell phenotypes, with the resulting inflammatory response elicited when such constructs are implanted in vivo.

## Conclusions

In this study, decellularized tendon and cartilage ECMs were solubilized either by pepsin digestion or urea extraction. The effects of these preparations on human MSC behavior were evaluated in 2D and 3D cultures. Pepsin-digested tendon and cartilage ECMs did not promote tissue-specific differentiation, as compared to controls, while urea-extracted fractions were mitogenic and upregulated homologous cell phenotypes. When MSCs were cultured as pellets, inhibition of endogenous TGF-β signaling using SB-431542 negated the tissue-specific inductivity of urea-extracted ECM, suggesting that endogenous TGF-β activity is necessary, but not sufficient, to explain the homologous bioactivity of tECM and cECM. When added as a component of a photocrosslinkable GelMA hydrogel, cECM independently upregulated early chondrogenesis of encapsulated MSCs and synergistically enhanced chondrogenesis when exogenous TGF-β was added as a medium supplement. Therefore, urea-extracted ECM fractions may be a promising biomaterial that, when combined with physically tunable scaffolds, can guide tissue-specific cell differentiation.

## Additional files

Additional file 1: Table S1. Target gene primer sequences for qPCR. (DOCX 14 kb)

Additional file 2: Table S2. Human growth factor array analysis of soluble tendon and cartilage ECM preparations. Growth factor concentrations (pg/mL) in 500 μg/mL soluble ECM preparations. (DOCX 15 kb)

Additional file 3: Figure S1. Effect of TGF-β signaling inhibition on biochemical content of MSC pellets. Medium conditions for pellet cultures were further supplemented with 10 μM SB-431542. Pellets supplemented with cECM exhibited elevated (albeit blunted) total sGAG and dsDNA contents compared to other medium conditions (*p* < 0.05, *n* = 9); dotted line indicates sGAG and dsDNA contents of control medium (without SB-431542). Statistically significant differences are indicated by overlying horizontal lines. (TIF 273 kb)

Additional file 4: Figure S2. Gene expression in cultures of MSCs seeded in 3D hydrogel derived from pepsin-digested ECM. MSCs were seeded in 5 mg/mL hydrogels of Collagen I (control), tAP, and cAP. ECM-derived hydrogels showed negligible tissue-specificity compared to controls. Statistically significant differences (*p* < 0.05, *n* = 9) are indicated by overlying horizontal lines. (TIF 408 kb)

Additional file 5: Figure S3. Macroscopic image of femoral condyles from young (6–8 weeks) and mature (2–3 years) cows. The osteochondral interface is distinct in adult animals but indistinct in young animals, with clear vasculature seen in dissected cartilage pieces. (TIF 1663 kb)

## Abbreviations

2D: Two-dimensional
3D: Three-dimensional
ACAN: Aggrecan
ALP: Alkaline phosphatase
ANOVA: Analysis of variance
bFGF: Basic fibroblast growth factor
BMP: Bone morphogenetic protein
BSA: Bovine serum albumin
cAP: Acid-pepsin digested cartilage extracellular matrix
cECM: Urea-extracted cartilage extracellular matrix
COL: Collagen
DAPI: 4’,6-Diamidino-2-phenylindole
DMEM: Dulbecco’s modified Eagle’s medium
DMF: Dimethylformamide
ECM: Extracellular matrix
EDTA: Ethylenediaminetetraacetic acid
FBS: Fetal bovine serum
GAPDH: Glyceraldehyde 3-phosphate dehydrogenase
GelMA: Methacrylated gelatin
H&E: Hematoxylin and eosin
HBSS: Hanks buffered salt solution
ITS: Insulin-transferrin-selenium
LAP: Lithium phenyl-2,4,6-trimethylbenzoylphosphinate
MKX: Mohawk
MSC: Mesenchymal stem cell
OCN: Osteocalcin
PBS: Phosphate-buffered saline
PCL: Poly-ε-caprolactone
PMSF: Phenylmethylsulfonyl fluoride
PSF: Penicillin-streptomycin-fungizone
qPCR: Quantitative real-time polymerase chain reaction
RUNX2: Runt-related transcription factor 2
SCX: Scleraxis
sGAG: Sulfated glycosaminoglycan
SOX9: SRY-box 9
tAP: Acid-pepsin digested tendon extracellular matrix
tECM: Urea-extracted tendon extracellular matrix
TGF-β: Transforming growth factor beta
THF: Tetrahydrofuran
TNC: Tenascin C
Tnmd: Tenomodulin
