# Supplementary material for: Tissue-specific bioactivity of soluble tendon-derived and cartilage-derived extracellular matrices on adult mesenchymal stem cells
Source: Stem Cell Res Ther. 2017 Jun 5;8:133. doi: 10.1186/s13287-017-0580-8 (PMC5460492; doi:10.1186/s13287-017-0580-8)
Supplement: Supplementary file 1 — Target gene primer sequences for qPCR. (DOCX 14 kb) [file 13287_2017_580_MOESM1_ESM.docx]

**Table S1**. Target gene primer sequences for qPCR.

| Gene | Primer sequence (5’🡪3’) | | Product size (bp) |
| --- | --- | --- | --- |
| *GAPDH* | Forward | CAAGGCTGAGAACGGGAAGC | 194 |
|  | Reverse | AGGGGGCAGAGATGATGACC |  |
| *18S rRNA* | Forward | GTAACCCGTTGAACCCCATT | 151 |
|  | Reverse | CCATCCAATCGGTAGTAGCG |  |
| *SCX* | Forward | TGCGAATCGCTGTCTTTC | 91 |
|  | Reverse | GAGAACACCCAGCCCAAA |  |
| *MKX* | Forward | GCAGCCACAGAAGCCGA | 502 |
|  | Reverse | AAATCTGGCTGTCGAACGGT |  |
| *TNC* | Forward | TTCACTGGAGCTGACTGTGG | 223 |
|  | Reverse | TAGGGCAGCTCATGTCACTG |  |
| *COL3A1* | Forward | CAGCGGTTCTCCAGGCAAGG | 179 |
|  | Reverse | CTCCAGTGATCCCAGCAATCC |  |
| *SOX9* | Forward | CTGAGCAGCGACGTCATCTC | 72 |
|  | Reverse | GTTGGGCGGCAGGTACTG |  |
| *ACAN* | Forward | GCTACACTGGCGAGCACTGTAACAT | 287 |
|  | Reverse | GCGCCAGTTCTCAAATTGCATGGG |  |
| *COL2A1* | Forward | GGATGGCTGCACGAAACATACCGG | 157 |
|  | Reverse | CAAGAAGCAGACCGGCCCTATG |  |
| *COL10A1* | Forward | gtgttttacgctgaacgataccaa | 273 |
|  | Reverse | ACCTGGTTTCCCTACAGCTGATG |  |
| *RUNX2* | Forward | CAACCACAGAACCACAAGTGCG | 196 |
|  | Reverse | TGTTTGATGCCATAGTCCCTCC |  |
| *ALP* | Forward | TGGAGCTTCAGAAGCTCAACACCA | 413 |
|  | Reverse | ATCTCGTTGTCTGAGTACCAGTCC |  |
| *OCN* | Forward | ATGAGAGCCCTCACACTCCTC | 294 |
|  | Reverse | GCCGTAGAAGCGCCGATAGGC |  |
| *COL1A1* | Forward | TAAAGGGTCACCGTGGCT | 355 |
|  | Reverse | CGAACCACATTGGCATCA |  |
