# Supplementary material for: Tissue-specific bioactivity of soluble tendon-derived and cartilage-derived extracellular matrices on adult mesenchymal stem cells
Source: Stem Cell Res Ther. 2017 Jun 5;8:133. doi: 10.1186/s13287-017-0580-8 (PMC5460492; doi:10.1186/s13287-017-0580-8)
Supplement: Supplementary file 2 — Human growth factor array analysis of soluble tendon and cartilage ECM preparations. Growth factor concentrations (pg/mL) in 500 μg/mL soluble ECM preparations. (DOCX 15 kb) [file 13287_2017_580_MOESM2_ESM.docx]

**Table S2**. Human growth factor array analysis of soluble tendon and cartilage ECM preparations. Growth factor concentrations (pg/mL) in 500 μg/mL soluble ECM preparations

|  |  |  |  | **6-8 weeks** | **2-3 years** |
| --- | --- | --- | --- | --- | --- |
| **Protein** | **tAP** | **tECM** | **cAP** | **cECM** | **cECM** |
| AR | 0.0 | 0.0 | 0.0 | **1.7** | 0.0 |
| BDNF | 0.0 | 0.0 | 0.0 | **2.0** | 0.0 |
| bFGF | 0 | **469.4** | 0.0 | **17,314.6** | **4,330.6** |
| BMP-4 | 0.0 | 0.0 | 0.0 | 0.0 | 0.0 |
| BMP-5 | 0.0 | 0.0 | 0.0 | **906.2** | 0.0 |
| BMP-7 | 0.0 | 0.0 | 0.0 | **170.7** | **57.1** |
| b-NGF | 0.0 | **0.1** | 0.0 | 0.0 | 0.0 |
| EGF | 0.0 | **0.1** | 0.0 | 0.0 | 0.0 |
| EGF R | **3.1** | 0.0 | **1.3** | 0.0 | 0.0 |
| EG-VEGF | 0.0 | 0.0 | 0.0 | **11.9** | **2.9** |
| FGF-4 | **114.9** | **137.3** | 0.0 | **276.1** | **79.3** |
| FGF-7 | 0.0 | 0.0 | 0.0 | 0.0 | 0.0 |
| GDF-15 | 0.0 | 0.0 | 0.0 | 0.0 | 0.0 |
| GDNF | 0.0 | 0.0 | 0.0 | 0.0 | 0.0 |
| GH | 0.0 | 0.0 | 0.0 | **31.4** | 0.0 |
| HB-EGF | 0.0 | 0.0 | 0.0 | 0.0 | 0.0 |
| HGF | **5.7** | 0.0 | 0.0 | 0.0 | **0.5** |
| IGFBP-1 | 0.0 | 0.0 | 0.0 | **24.8** | 0.0 |
| IGFBP-2 | 0.0 | 0.0 | 0.0 | **1,952.7** | **114.0** |
| IGFBP-3 | 0.0 | **162.8** | 0.0 | **260.3** | **233.7** |
| IGFBP-4 | 0.0 | 0.0 | 0.0 | **58.7** | **0.1** |
| IGFBP-6 | 0.0 | 0.0 | 0.0 | **80.1** | 0.0 |
| IGF-1 | 0.0 | 0.0 | 0.0 | 0.0 | 0.0 |
| Insulin | **18.4** | 0.0 | **70.7** | **425.8** | **149.6** |
| MCSF R | 0.0 | 0.0 | 0.0 | 0.0 | 0.0 |
| NGF R | 0.0 | 0.0 | 0.0 | **11.7** | 0.0 |
| NT-3 | **1.3** | **4.9** | **0.6** | **47.8** | **22.0** |
| NT-4 | 0.0 | **2.2** | 0.0 | **22.3** | 0.0 |
| OPG | 0.0 | **0.1** | 0.0 | **2.9** | **153.3** |
| PDGF-AA | 0.0 | 0.0 | 0.0 | 0.0 | 0.0 |
| PIGF | 0.0 | 0.0 | 0.0 | 0.0 | 0.0 |
| SCF | **1.4** | **1.9** | 0.0 | **1.4** | **1.3** |
| SCF R | 0.0 | **15.7** | 0.0 | **16.5** | **10.0** |
| TGFa | 0.0 | 0.0 | 0.0 | 0.0 | 0.0 |
| TGFb1 | **83.2** | **872.2** | **942.7** | **2,789.5** | **758.0** |
| TGFb3 | 0.0 | **34.6** | 0.0 | **154.7** | 0.0 |
| VEGF | 0.0 | 0.0 | 0.0 | 0.0 | 0.0 |
| VEGF R2 | 0.0 | 0.0 | 0.0 | 0.0 | 0.0 |
| VEGF R3 | 0.0 | 0.0 | 0.0 | **8.0** | 0.0 |
| VEGF-D | 0.0 | 0.0 | 0.0 | 0.0 | 0.0 |
