# Supplementary figures and images for: Tissue-specific bioactivity of soluble tendon-derived and cartilage-derived extracellular matrices on adult mesenchymal stem cells
Source: Stem Cell Res Ther. 2017 Jun 5;8:133. doi: 10.1186/s13287-017-0580-8 (PMC5460492; doi:10.1186/s13287-017-0580-8)

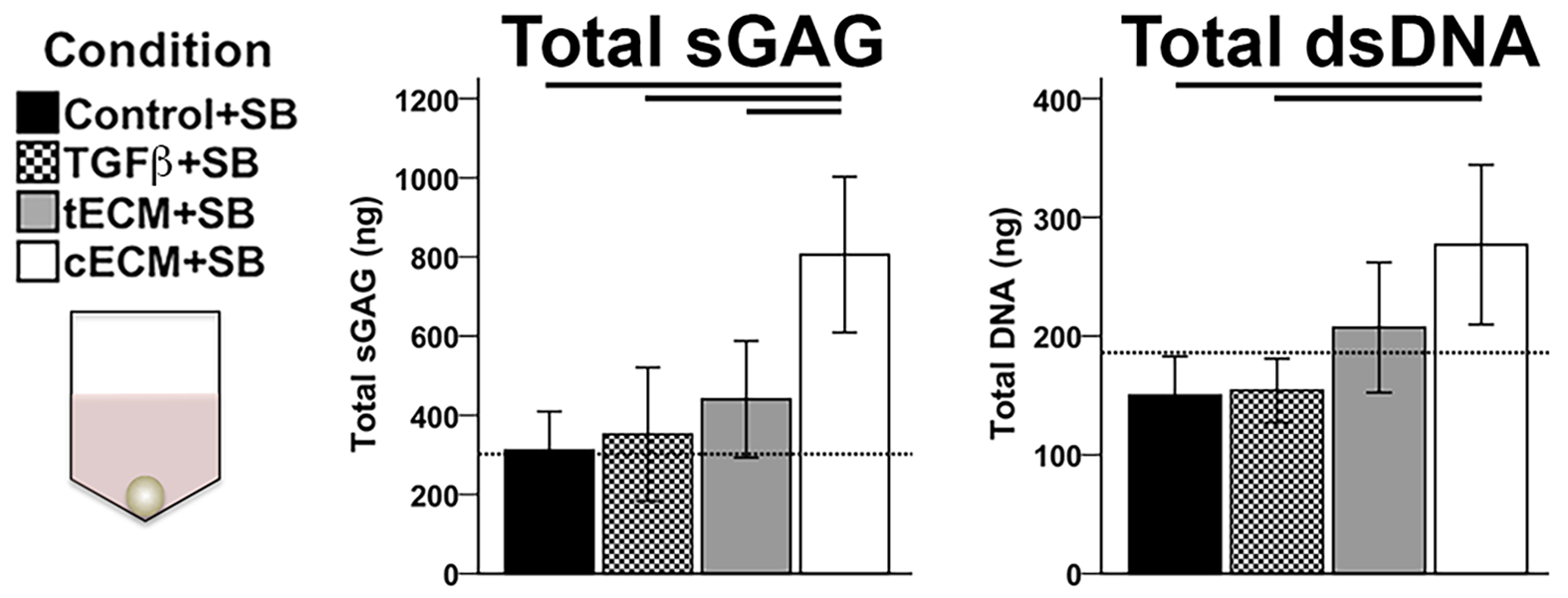

Supplement: Supplementary file 3 — Effect of TGF-β signaling inhibition on biochemical content of MSC pellets. Medium conditions for pellet cultures were further supplemented with 10 μM SB-431542. Pellets supplemented with cECM exhibited elevated (albeit blunted) total sGAG and dsDNA contents compared to other medium conditions (p < 0.05, n = 9); dotted line indicates sGAG and dsDNA contents of control medium (without SB-431542). Statistically significant differences are indicated by overlying horizontal lines. (TIF 273 kb) [file 13287_2017_580_MOESM3_ESM.tif]

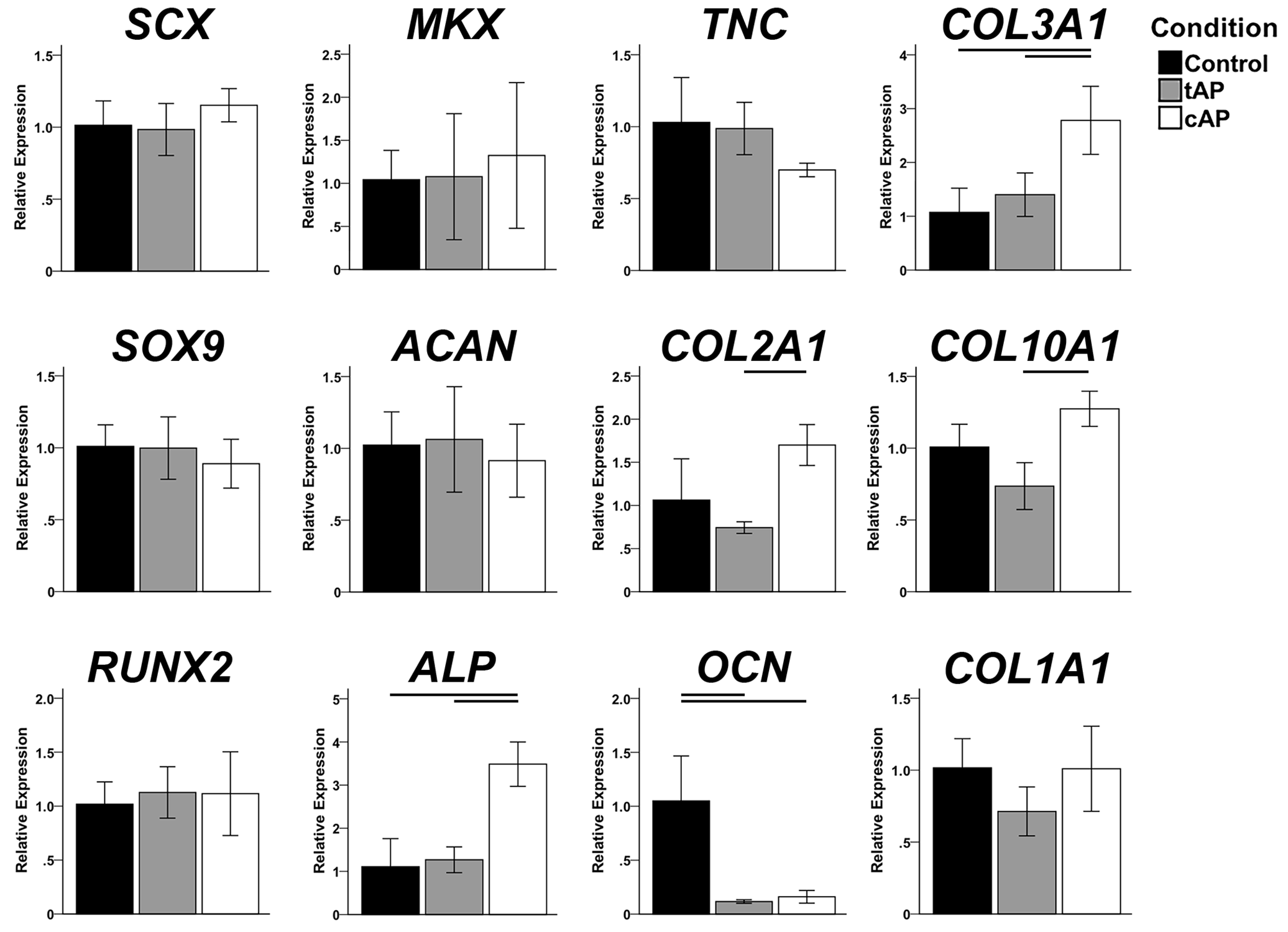

Supplement: Supplementary file 4 — Gene expression in cultures of MSCs seeded in 3D hydrogel derived from pepsin-digested ECM. MSCs were seeded in 5 mg/mL hydrogels of Collagen I (control), tAP, and cAP. ECM-derived hydrogels showed negligible tissue-specificity compared to controls. Statistically significant differences (p < 0.05, n = 9) are indicated by overlying horizontal lines. (TIF 408 kb) [file 13287_2017_580_MOESM4_ESM.tif]

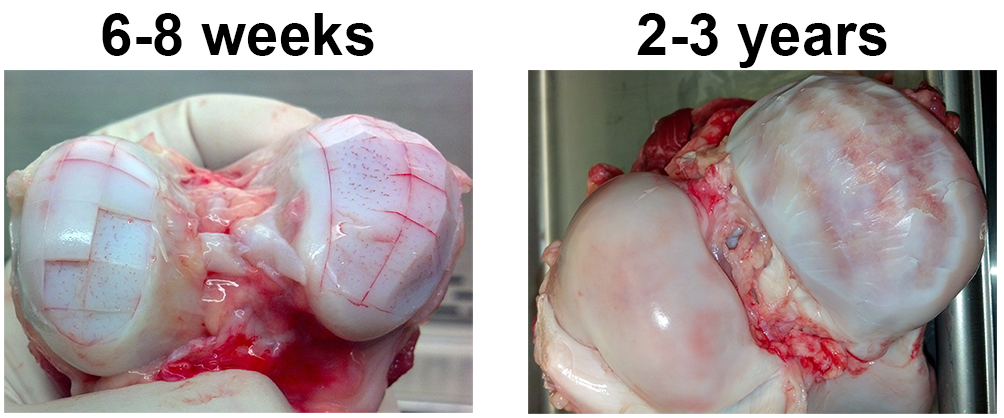

Supplement: Supplementary file 5 — Macroscopic image of femoral condyles from young (6–8 weeks) and mature (2–3 years) cows. The osteochondral interface is distinct in adult animals but indistinct in young animals, with clear vasculature seen in dissected cartilage pieces. (TIF 1663 kb) [file 13287_2017_580_MOESM5_ESM.tif]
